# Co-Inhibition of the Immunoproteasome Subunits LMP2 and LMP7 Ameliorates Immune Thrombocytopenia

**DOI:** 10.3389/fimmu.2020.603278

**Published:** 2021-01-20

**Authors:** Sheng-hong Du, Yu-jiao Xiang, Lu Liu, Mu Nie, Yu Hou, Ling Wang, Ban-ban Li, Miao Xu, Qing-liang Teng, Jun Peng, Ming Hou, Yan Shi

**Affiliations:** ^1^Department of Hematology, Qilu Hospital, Cheeloo College of Medicine, Shandong University, Jinan, China; ^2^Department of Hematology, Taian Central Hospital, Taian, China; ^3^Division of Immunology and Allergy, Department of Medicine Solna, Karolinska Institutet, Karolinska University Hospital, Stockholm, Sweden; ^4^Shandong Provincial Key Laboratory of Immunohematology, Qilu Hospital, Cheeloo College of Medicine, Shandong University, Jinan, China; ^5^Shandong Provincial Clinical Research Center in Hematological Diseases, Jinan, China; ^6^Leading Research Group of Scientific Innovation, Department of Science and Technology of Shandong Province, Qilu Hospital, Cheeloo College of Medicine, Shandong University, Jinan, China

**Keywords:** immune thrombocytopenia, immunoproteasome, LMP2, LMP7, FcγRs, T cell, treatment

## Abstract

The immunoproteasome, a special isoform of the 20S proteasome, is expressed when the cells receive an inflammatory signal. Immunoproteasome inhibition proved efficacy in the treatment of autoimmune diseases. However, the role of the immunoproteasome in the pathogenesis of immune thrombocytopenia (ITP) remains unknown. We found that the expression of the immunoproteasome catalytic subunit, large multifunctional protease 2 (LMP2), was significantly upregulated in peripheral blood mononuclear cells of active ITP patients compared to those of healthy controls. No significant differences in LMP7 expression were observed between patients and controls. ML604440, an specific LMP2 inhibitor, had no significant impact on the platelet count of ITP mice, while ONX-0914 (an inhibitor of both LMP2 and LMP7) increased the number of platelets. *In vitro* assays revealed that ONX-0914 decreased the expression of FcγRI in ITP mice and decreased that of FcγRIII in ITP patients, inhibited the activation of CD4^+^ T cells, and affected the differentiation of Th1 cells in patients with ITP. These results suggest that the inhibition of immunoproteasome is a potential therapeutic approach for ITP patients.

## Introduction

Immune thrombocytopenia (ITP) is an autoimmune bleeding disorder, characterized by persistent thrombocytopenia in which the destruction of autoantibody-opsonized platelets by phagocytic cells plays an important role. As an acquired autoimmune disease, the immune response underlying ITP pathogenesis involves a complex interaction between antigen-presenting cells (APCs), T cells, and B cells, in which the recognition of platelet antigens by autoreactive T helper (Th) cells, as well as Th cell activation, are critical events. In addition to activating B cells, which leads to the secretion of pathogenic autoantibodies, activated Th cells promote the production of cytokines. Recent investigations have substantiated a Th1 polarization of the immune response in ITP (1, 2).

The proteasome is a multi-subunit proteolytic complex that degrades the majority of non-lysosomal proteins in eukaryotic cells. The catalytic activity of proteasomes is exerted by three subunits, β1, β2, and β5, which are constitutively expressed in all cells (3). In cells treated with interferon (IFN)-γ and tumor necrosis factor (TNF)-α, or in hematopoietic cells, these subunits are replaced by the inducible subunits of low-molecular mass polypeptide (LMP)2 (β1i), multicatalytic endopeptidase complex-like (MECL)-1 (β2i), and LMP7 (β5i), forming an immunoproteasome (3, 4). The immunoproteasomes have higher chymotrypsin-like activity and a higher ability to process and present antigens, as compared to standard proteasomes (5).

Increasing evidence has indicated that the immunoproteasome is associated with the adaptive immune response and, recently, it has been shown to be involved in the pathogenesis of autoimmune diseases (6–11) and tumors (12–14). Immunoproteasome inhibition prevents chronic antibody-mediated allograft rejection in renal transplantation (15, 16), and promotes long-term cardiac allograft acceptance in mice. In another study, application of selective immunoproteasome-subunits inhibitors may allow for the targeted treatment of cells featuring high immunoproteasome expression levels as in (malignant) hematopoietic cells. Due to this, clinical application of immunoproteasome inhibitors could lower side effects of proteasome inhibition and improve future therapy options (6). In this study, increased LMP2 expression was observed in PBMCs from active ITP patients compared to healthy controls. However, inhibiting LMP2 alone failed to restore platelet counts, whereas the dual inhibition of LMP2 and LMP7 alleviated thrombocytopenia in passive murine models of ITP. Combined LMP2 and LMP7 inhibition not only attenuated macrophage phagocytosis of antibody-coated platelets by decreasing the expression of FcγRI and FcγRIII in ITP mice or patients, but also inhibited the activation of CD4^+^ T cells and the secretion of Th1 cytokines in patients with ITP. Our results suggest that the immunoproteasome may play a pathogenic role in ITP, and demonstrate that the co-inhibition of LMP2 and LMP7 is a novel potential therapeutic strategy for the treatment of ITP.

## Materials and Methods

### Patients and Controls

Thirty-three patients (19 females and 14 males; age range 15–74 years, median 43 years; platelet count range 0–26 × 10^9^/L, median 8 × 10^9^/L) were enrolled in this study. None of the patients had received any ITP-specific therapy for at least one month (**Table 1**). All patients were diagnosed and selected according to the primary ITP criteria from the International Working Group consensus report, and other diseases causing thrombocytopenia were excluded (17). The healthy control group included thirty healthy adult volunteers (14 females and 16 males; age range 21–45 years, median 30 years; platelet count range 115–290 × 10^9^/L, median 198 × 10^9^/L). The enrolment took place between November 2017 and November 2019 at the Department of Hematology, Qilu Hospital, Shandong University and at the Department of Hematology, Jinan Central Hospital affiliated with Shandong University. This study was approved by the Medical Ethical Committee of Qilu Hospital, Shandong University, and by the Jinan Central Hospital affiliated with Shandong University. The study was conducted in accordance with the Declaration of Helsinki.

**Table 1:** ITP patient characteristics.

Patient#	Age(yrs)	Sex	Platelet count(10^9^/L)	Major previous drugs
1	59	F	1	Dex, DAC
2	62	F	8	Dex, TPO, DAC
3	68	M	26	Dex
4	32	F	3	Dex, DAC
5	19	F	19	Dex, RTX, TPO
6	72	M	1	Dex, RTX, TPO
7	52	M	5	Dex
8	27	M	0	Dex, IVIg, TPO, DAC
9	31	F	24	Dex, RTX, TPO
10	64	M	20	Dex, TPO
11	25	F	2	Dex, TPO, DAC
12	15	F	10	Dex, RTX, TPO
13	59	F	2	Dex, RTX, TPO
14	23	M	16	Dex, TPO
15	49	F	7	Pred, TPO
16	30	F	19	Dex, IVIg, RTX
17	36	F	4	Dex, TPO
18	52	F	7	Dex, hTPO
19	57	F	1	Pred, TPO
20	29	F	21	Pred, CTX, RTX, TPO
21	24	M	11	Dex, IVIg, TPO
22	48	M	21	Dex, TPO
23	25	F	2	Dex, TPO, DAC
24	52	M	5	Dex
25	56	F	4	Dex, RTX
26	71	M	9	Dex, RTX
27	60	F	1	Dex, RTX, DAC
28	52	M	2	DAC
29	20	F	16	Dex, IVIg, RTX
30	74	M	1	DAC
31	27	F	2	Dex
32	20	M	4	Dex
33	24	M	4	Pred, IVIg, TPO

CTX, cytoxan; DAC, Decitabine; Dex, dexamethasone; IVIg, intravenous gamma globulin; Pred, prednisone; RTX, rituximab; TPO, thrombopoietin.

### Proteasome Inhibitors

For all *in vitro* experiments, ML604440 (Probechem, PC-60968), ONX -0914 (MCE, HY-13207) were dissolved at a concentration of 10 mM in DMSO and stored at −80°C. A final DMSO concentration of 0.3% was used. For proteasome inhibition in mice, ML604440 was diluted in PBS with 5% polyethylene glycol (PEG-400; Sangon Biotech) and 1% Tween-80 (Sangon Biotech) immediately before use. ONX 0914 was formulated in an aqueous solution of 10% (w/v) sulfobutylether-beta-cyclodextrin and 10 mM sodium citrate (pH 6) and administered to mice as a subcutaneous bolus dose of 10 mg/kg.

### Primary Human Cell Preparation

Peripheral blood was collected into EDTA-anticoagulant vacutainer tubes. Peripheral blood mononuclear cells (PBMCs) were isolated using Ficoll Hypaque centrifugation (Amersham Biosciences, Piscataway, NJ, USA) and washed twice with 0.9% saline by centrifugation at 300 ×g for 10 min. To isolate circulating CD14+ monocytes or CD4+T cells, the PBMCs were resuspended in AutoMACS sample buffer (Miltenyi Biotec, Bergisch Gladbach, Germany). Anti-CD14-coated magnetic beads or anti- CD4-coated magnetic beads (10 μL per 10^7^ cells, Miltenyi Biotec) were added with constant rotation (15 min, 4°C), washed, and purified by Miltenyi Biotec column separation. The purity of isolated cells was > 90%, as assessed by flow cytometry.

### Analysis of LMP2 and LMP7 Expression by ELISA

PBMCs of ITP patients and healthy controls were resuspend in cell lysis buffer (Cell Signaling) at a concentration of 10^7^ cells/mL. Phenylmethylsulfonyl fluoride (PMSF, Cell Signaling) was routinely added as a supplement to lysis buffers immediately before lysis. When necessary, the cells were subjected to ultrasonication until the solution was clarified. The cells were centrifuged at 1,500×g for 10 min at 2–8°C to remove cellular debris. LMP2 and LMP7 levels were measured using commercial Quantikine enzyme-linked immunosorbent assay (ELISA) kits (Biomatik, EKU05564, EKU09068, Human) according to the manufacturer’s instructions.

### Analysis of LMP2 and LMP7 Expression by RT-PCR

RNAiso Plus (Takara, Japan) was used to isolate total RNA from the PBMCs of ITP patients and healthy controls. RNA was reversely transcribed into cDNA using the PrimeScript RT reagent kit (Perfect Real Time; Takara) according to the manufacturer’s instructions. Quantitative real-time PCR (q-PCR) was performed for LMP2, LMP7, and the endogenous control GAPDH on a LightCycler^®^ 480 System (Roche Applied Science, Mannheim, Germany). Primers and conditions are reported in detail in **Table 2**. To calculate relative changes in gene expression, the target genes were compared to GAPDH using the comparative delta Ct (△△Ct) method.

**Table 2:** Primers for q-PCR.

	Forward	Reverse
Homo-LMP2(PSMB9)	TGCTGACTCGACAGCCTTTT	GCCCAAGATGACTCGATGGT
Homo-LMP7(PSMB8)	CTACGGGGTCATGGACAGTG	GCATAAGCAATAGCCCTGCG

### Passive ITP Model

To establish the passive ITP mouse model, male C57BL/6J mice (6-8-wks old, platelet count 1,020–1,880 × 10^9^/L, median 1370 × 10^9^/L) were intraperitoneally injected with anti-platelet monoclonal antibody (mAb, rat anti-mouse CD41, clone MWReg30; BD Biosciences) at an initial dose of 0.375 mg/kg body weight and follow-up doses of 0.125 mg/kg every 36 h. ML604440 (10 mg/kg) or ONX-0914 (10 mg/kg) were injected intraperitoneally every day to passive ITP mice simultaneously with the first intravenous injection of anti-platelet monoclonal antibody.

To analyze platelet counts, whole blood samples (5 μL) were collected from the vein of lower extremities and mixed with anticoagulant ACD solution (38 mM citric acid, 75 mM sodium citrate, 100 mM dextrose). Platelets were counted at 0, 24, 48, 72, 120, and 168 h after the first anti-CD41 injection with a hematology analyzer (KX-21N; Sysmex). The optimal mAb dosage was determined according to the platelet count. The time for platelet recovery in the passive ITP mice model was 5 to 8 days without treatment (18, 19). ITP mice were euthanized on day 7, and spleens, thymus and blood were gathered. Single cell suspensions were prepared. Cells were treated with hemolysis buffer to remove red blood cells. After washing in PBS, we counted cells and split them for flow cytometry staining.

### Cell Surface Staining for Flow Cytometric Determination of FcγRs

For analysis of FcγR expression in macrophages, 1 × 10^6^ cells from the spleen of ITP mice were stained with anti-mouse CD11b-FITC (Biolegend), anti-mouse CD16/32-PE (Biolegend), and anti-mouse CD64 (Biolegend) monoclonal antibodies for 30 min at 37°C in the dark. Purified human CD14+ cells were exposed (continuous treatment) to DMSO or ML604440 (300 nM) or ONX-0914 (30 nM) for 24 h. Cultured human monocytes/macrophages (1 × 10^6^) were incubated (RT, 30 min in the dark) with a cocktail of anti-human CD14-APC (Biolegend), anti-human CD16-FITC (Invitrogen), and anti-human CD64-PE (Biolegend) antibodies. Data analysis was carried out using a FACS Calibur flow cytometer equipped with Kaluza Flow Cytometry Analysis Software (Beckman Coulter).

### Preparation of Platelets

Platelet isolation and labelling with 5-chloromethylfluorescein diacetate (CMFDA, Invitrogen) for phagocytic assays were carried out as previously described (20–22). Briefly, trisodium citrate-anticoagulant venous peripheral blood from healthy volunteers was centrifuged (120 ×g, 10 min) to obtain platelet-rich plasma. The platelets were then isolated by centrifugation (800 ×g, 5 min), and adjusted to 10^9^/mL with 5 μM prostaglandin E1 (Cayman Chemical). The platelets were incubated with CMFDA (final concentration, 10 μM; 30 min at 37°C in the dark), washed, and resuspended in PBS. For opsonization, CMFDA-labeled platelets were incubated with 10 μg/mL anti-human CD41 mAbs (eBioscience; 30 min, room temp) and washed once before the phagocytic assay.

### Phagocytosis Assay

CMFDA-labelled platelets were phagocytosed by monocytes/macrophages according to previously reported methods with mild modifications (20–22). Monocytes/macrophages (1 × 10^6^/well) were treated with DMSO (control), or ML604440 (300 nM) or ONX-0914 (30 nM) for 24 h, incubated with 50 ng/mL phorbol 12-myristate 13-acetate (PMA; Multisciences, Hangzhou, China) for 1 h, and washed twice with PBS. Opsonized platelets were then incubated with monocytes/macrophages (50:1; 1 h, 37°C or 4°C as a control), and washed with PBS to remove unbound platelets. The adherent macrophages were removed with a scraper and stained with anti-CD61-APC (Invitrogen). The fluorescence intensity was determined by flow cytometry. The mean fluorescence intensity (MFI) of CD61-APC-negative and CMFDA-positive cells represented platelet engulfment by macrophages. The phagocytic index was calculated by the formula: MFI obtained at 37°C)/MFI obtained at 4°C.

### Effects of Immunoproteasome Inhibitors on T Activation and Cell Differentiation

To investigate T cell activation, PBMCs (1 × 10^6^ cells/ml) were preincubated with DMSO or ML604440 (300 nM) or ONX-0914 (30 nM) for 2h and were seeded in 96-well microplates coated with anti-CD3/CD28 antibodies. After treated for 10h, the cells were harvested and incubated with CD69-PE(Biolegend), CD4-APC (Biolegend). In parallel, the cells were harvested after 72h to measure the expression of CD25 by incubating with CD25-APC (Biolegend)and CD4-FITC (Biolegend) in the dark at Room temperature(RT) for 30 min, washed with cold PBS, and analyzed by flow cytometry within 1 h.

Magnetically purified CD4^+^ T cells (MACS; Miltenyi Biotec) were exposed (continuous treatment) to DMSO, ML604440 (300 nm) or ONX-0914 (30 nM) for 2 h before stimulation with plate-bound antibodies against CD3 and CD28 (Biolegend) for 3 days. The intracellular expression of IL-17A and IFN-γ in CD3^+^CD8^-^ cells (CD3-PE/cy7, clone SK3; CD8-APC-eFluor780, clone RPA-78, eBioscience) was measured by using the appropriate antibodies (clone eBio64Cap17 and clone 4SB3; eBioscience) 4 h after exposure to 50 ng/ml PMA (Sigma) and 500 ng/mL ionomycin (Sigma) in the presence of 10 µg/mL brefeldin A (Sigma) by flow cytometry (Accuri C6; BD Biosciences).

### Western Blot

PBMCs (2 × 10^7^ cells/sample) were lysed in radio-immunoprecipitation assay (RIPA) buffer (Bestbio, Shanghai, China). Immunoblots were performed using polyclonal rabbit anti-human/mouse LMP2 (ab242061, Abcam), LMP7 (ab180606, Abcam), LMP10 (ab183506, Abcam), β-actin (ab179467, Abcam), GAPDH (ab181602, Abcam), p-STAT1 (Cell Signaling), STAT1 (Cell Signaling), p-STAT3 (Cell Signaling), and STAT3 (Cell Signaling) antibodies. Semi-quantitative evaluation of protein levels was performed by comparing band intensity between the protein of interest and the housekeeping controls, GAPDH or β-actin. The level of phosphorylated proteins was measured based on the ratio of phosphorylated protein to total protein.

### Statistical Analysis

All data were analyzed by SPSS software and are presented as mean ± SD. Two data sets were analyzed using t-test or paired t-test for matched observations. Multiple groups were compared using ANOVA. All data are presented as mean ± standard error of the mean (SEM). P < 0.05 were considered statistically significant.

## Results

### LMP2 and LMP7 in Active ITP Patients

**Figure 1:**
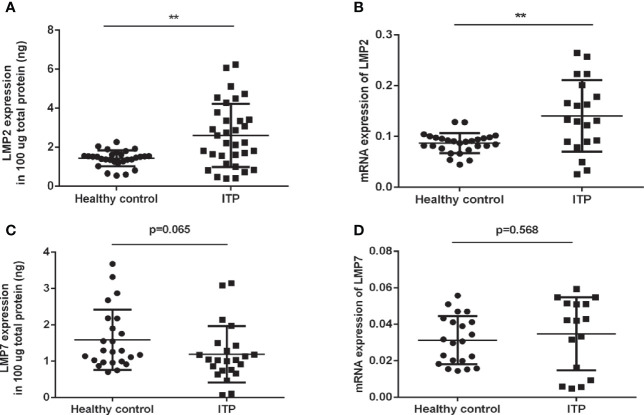
LMP2 protein and mRNA expression are upregulated in ITP patients. **(A)** Immunosubunit LMP2 protein expression in ITP patients (n=33) and healthy controls (n=29) were assayed by ELISA. **(B)** LMP2 mRNA expression in ITP patients (n=20) and healthy controls (n=27) were determined by qPCR. **(C)** Immunosubunit LMP7 protein expression in ITP patients (n=23) and healthy controls (n=24) were assayed by ELISA. **(D)** LMP7 mRNA expression in ITP patients (n=16) and healthy controls (n=21) were determined by qPCR. Differences between patients and controls were compared by a Student-Newman-Keuls test. Bars represent SD; **P < 0.01.

To investigate whether the immunoproteasome plays a role in human ITP, we compared the levels of LMP2 and LMP7 in the PBMCs of ITP patients (**Table 1**) and healthy individuals. ELISA and RT-PCR analysis showed that LMP2 protein expression and gene transcription were both significantly upregulated in ITP patients compared to healthy controls (**Figures 1A, B**). However, no differences in LMP7 expression were found in the PBMCs of patients and healthy controls (**Figures 1C, D**). These results suggested that LMP2 was upregulated in the PBMCs of ITP patients and could play a role in the pathogenesis of ITP.

### Co-Inhibition of the Immunoproteasome Subunits LMP2 and LMP7 Alleviate Thrombocytopenia in ITP Mice

**Figure 2:**
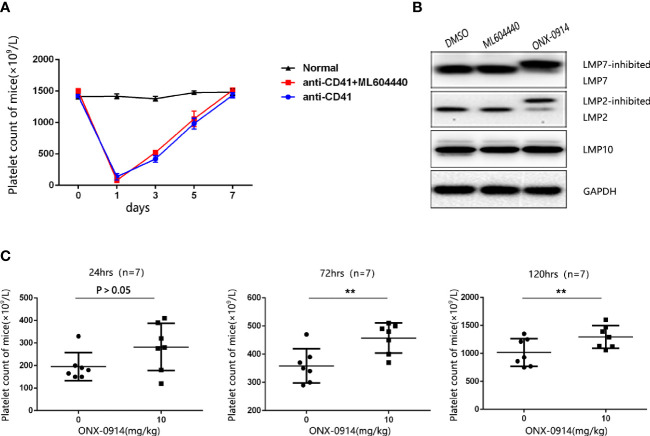
ONX-0914 but not ML604440 reversed thrombocytopenia in ITP mice. **(A)** ML604440 (10 mg/kg) everyday did not significantly improve platelet counts in mice immunized by monoclonal rat anti-mouse CD41 platelet antibody. **(B)** Altered electrophoretic mobility of IP subunits by covalent modification with ONX 0914. Shown are representative Western blots out of three independent experiments with similar outcome. **(C)** ONX-0914 (10 mg/kg) everyday improved platelet counts in mice at 24 hours, 72 hours and 120 hours after immunization. **P < 0.01.

To determine the role of LMP2 in ITP, we used the passive murine ITP model. The passive murine ITP model was elicited by intraperitoneally injected with anti-platelet monoclonal antibody (mAb, rat anti-mouse CD41) at an initial dose of 0.375 mg/kg body weight and follow up doses of 0.125 mg/kg every 36 hours. Mice were treated daily with 10mg/kg ML604440, a dose which inhibits LMP2 *in vivo* (**Supplementary Figure 2A**). No significant changes in platelet counts were observed in mice treated with ML604440 (10mg/kg) compared to vehicle-treated mice (**Figure 2A**). ONX-0914 was previously described as a selective LMP7 inhibitor. Our data and a previous study demonstrated that prolonged exposure of cells to ONX 0914 leads to inhibition of both LMP7 and LMP2. ONX-0914 was frequently used at a concentration of 300 nM in cells (23). Our research confirmed that 300 nmol/L ONX-0914 for 3 days induced cell apoptosis in human cells (**Supplementary Figure 1B**). Hence, we reduced the concentration of ONX-0914 to 30nM, which can well inhibit LMP2 and LMP7 and had no cytotoxicity to cells (**Figure 2B**, **Supplementary Figure 1B**). The treatment of passive ITP mice with 10mg/kg ONX-0914 increased the platelet count at 24, 72, and 120 h after immunization (**Figure 2C**), suggesting that ONX-0914 had therapeutic effects on ITP.

### Effect of ONX-0914 Treatment on the Expression of FcγRs in the Monocytes of ITP Mice and Patients

**Figure 3:**
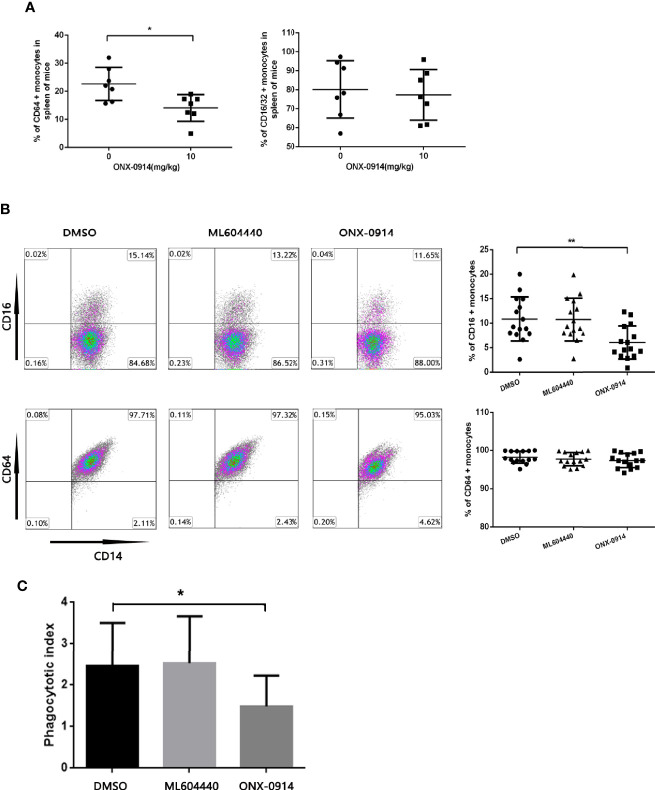
ONX-0914 decreased activating FcγRs of monocytes in ITP mice and patients, inhibited the phagocytosis of macrophages *in vitro*. **(A)** Flow cytometry analysis of FcγRI (CD64) or FcγRIII/FcγRII (CD16/32) expression on monocytes of passive ITP mice spleen. **(B)** Flow cytometry analysis of FcγRI (CD64) or FcγRIII (CD16) expression on monocyte-derived macrophage of patients. **(C)** Phagocytic capacity of macrophages from ITP patients after exposure to ML604440 (300 nM) or ONX-0914 (30 Nm) or vehicle. *P < 0.05, **P < 0.01.

To investigate the impact of ONX-0914 on FcγRs regulation, the surface expression of FcγRs in the monocytes of spleen from ITP mice was determined. In spleen monocytes/macrophages of ITP mice, the level of FcγRI (CD64) decreased dramatically after ONX-0914 administration (n = 7, P<0.01). On the other hand, FcγRIII/II (CD16/32) expression was not substantially affected under these conditions (**Figure 3A**).

To study the effect of ONX-0914 on patient monocytes, purified human CD14+ cells were incubated with DMSO, ML604440 (300 nM) or ONX-0914 (30 nM) for 24 h. In the monocytes of ITP patients treated with ONX-0914, FcγRIII (CD16) expression was found to be significantly decreased compared to DMSO-treated controls (P<0.01), while these treatments had no significant impact on FcγRI (CD64) expression (**Figure 3B**).

To evaluate whether the shift in the FcγR status of monocytes was associated with functional changes, isolated monocytes were treated with DMSO, ML604440 (300 nM) or ONX-0914 (30 nM) before incubation with opsonized CMFDA-labelled platelets for phagocytosis assays. As expected, monocytes treated with ONX-0914 exhibited significantly lower phagocytic capacity compared to controls (P<0.05; **Figure 3C**).

### ONX-0914 Inhibited T Cell Activation

**Figure 4:**
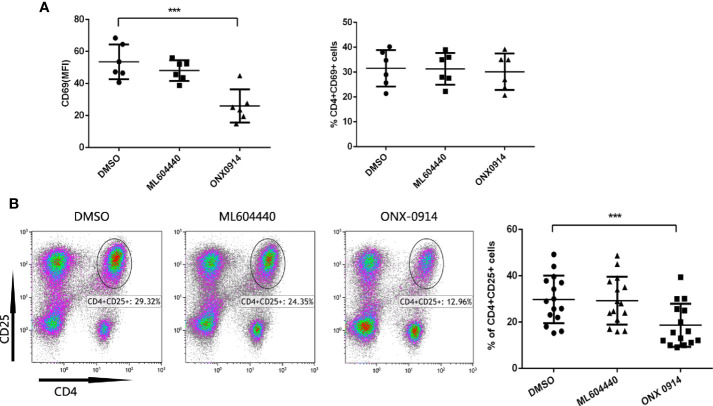
ONX-0914 decreased activation level of T cells from immune thrombocytopenia (ITP) patients. **(A)** Expression of CD69 in CD4+T cells was analyzed by flow cytometry. The PBMC cells in the presence/absence of ML604440 or ONX-0914 were co-stimulated with anti-CD3/CD28 antibodies for 10 h. Shown are the MFI (left) and %(right) of CD69. ONX-0914 (30 nM) reduced the MFI of CD16 cells in CD4+ T cells without influence of % CD4+CD16+ T cells. **(B)** Expression of CD25 in CD4+T cells was analyzed by flow cytometry. The PBMC cells in the presence/absence of ML604440 or ONX-0914 were co-stimulated with anti-CD3/CD28 antibodies for 72 h. ONX-0914 (30 nM) reduced the percentage and number of CD4^+^CD25^+^ cells in ITP patients than ML604440 (300 nM) and DMSO. ***P < 0.001.

To investigate whether ONX-0914 interferes with T cell activation events, expression of the T cell activation markers CD69 and CD25 in ITP patients T cells was assessed after co-stimulation with anti-CD3/CD28 antibodies. CD69 is an earlier and more sensitive marker of T cell activation (24–26). CD25 is a component of the IL-2 receptor and is involved in late-stage T cell proliferation and differentiation (27). After treated for 10 h, CD69 was assayed. Intriguingly, while the MFI (median fluorescent intensity) value of CD69 was decreased after ONX-0914 treatment, the percentage of CD69-positive cells was not altered under the same conditions(**Figure 4A**). CD25 was detected on 72h after stimulated. In CD4+ T cells of ITP patients, treatment with ONX-0914 for 72 h caused a significant decrease in CD25 expression, as compared to controls (p<0.0001, **Figure 4B**), while ML604440 had no significant effects. ONX-0914 also reduced CD4+CD25+ T cells in PBMCs and thymus of passive ITP mice (**Supplementary Figure 3**). This indicates that LMP2 and LMP7 controlled the activation of CD4+ T cells.

### ONX-0914 Suppressed Th1 Cell Differentiation

**Figure 5:**
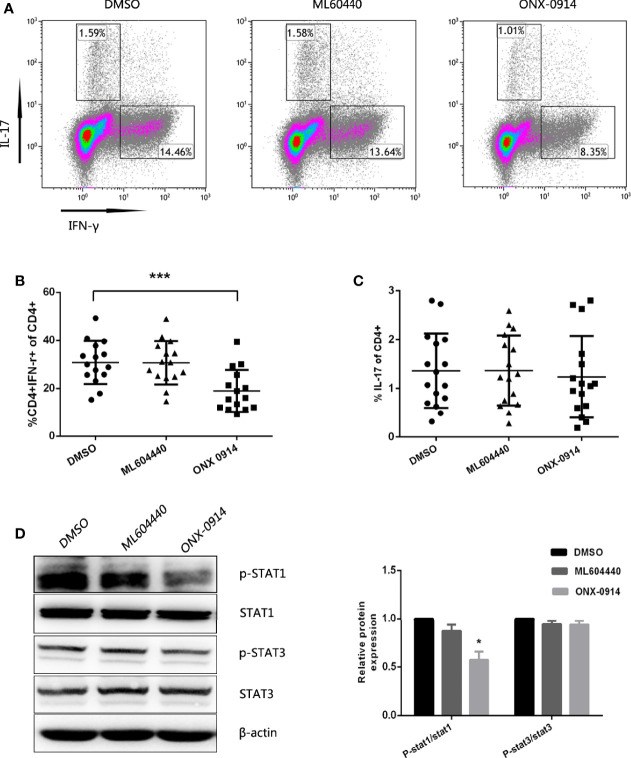
ONX-0914 suppress differentiation of Th1. **(A–C)** Differentiation was measured in 3 day cultures of CD4^+^ T cells isolated from ITP patients that were exposed to continuous DMSO, ML604440 (300 nM), or ONX 0914 (30 nM), and that were stimulated with platelet-bound antibodies to CD3/CD28. IFN-γ and IL-17 expression were detected by intracellular cytokine staining after 4 hours of restimulation with PMA/ionomycin/BFA. Values reflect percentage of CD4^+^ cells that were IFN-γ^+^ or IL-17A^+^. All data were statistically compared to DMSO-treated group. ***P < 0.001. One-way ANOVA. **(D)** Western blot of purified CD4^+^ T cells that were cultured for 3 days with anti-CD3/CD28 in the presence or absence of ML604440 (300 nM) or ONX 0914 (30 nM). Data are representative of at least three independent experiments. *P < 0.05 vs DMSO.

To examine the potential role of ONX-0914 in Th cell regulation, CD4+ T cells from ITP patients were magnetically sorted and cultured *in vitro* in the presence of DMSO, 300 nM ML604440 or 30 nM ONX 0914, and then stimulated with anti-CD3/CD28. After 3 days in culture, the number of Th1 cells was determined by intracellular IFN-γ staining of CD3+CD8- T cells and the number of Th17 cells was determined by intracellular IL-17A staining of CD3+CD8- T cells (**Figure 5A**). In the presence of ONX-0914, the percentage of IFN-γ-producing CD4+ T cells was lower compared to cells treated with DMSO. Notably, CD4+ T cells simultaneously exposed to a specific LMP2 inhibitor (ML604440) had no influence on Th1 polarization (**Figure 5B**). No significant inhibition of Th17 differentiation was observed after treatment with either ML604440 or ONX-0914 (**Figure 5C**).

We then explored whether ONX-0914 affected the phosphorylation of STAT1 in CD4+ T cells. PBMCs pulsed with ONX-0914 exhibited a lower level of STAT1 phosphorylation compared to the untreated control or ML604440. However, ONX-0914 or ML604440 had no significant effects on phosphorylation of STAT3 and STAT3 (**Figure 5D**).

## Discussion

The proteasome is part of the ubiquitin-proteasome system (UPS), which is responsible for degrading damaged or misfolded proteins tagged with ubiquitin. The immunoproteasome is an alternative form of the constitutive proteasome, which comprises three subunits, namely, LMP2 (β1i), LMP7 (β5i), and LMP10 (β2i) (28). Recent studies have demonstrated that the immunoproteasome participates in several experimentally induced inflammatory and autoimmune diseases (6–11). We and other groups demonstrated that IFN-ɣ is significantly increased in ITP patients (29, 30). Cells exposure to IFN-ɣ resulted in the expression of immunosubunits, which may explain the upregulation of LMP2 protein and mRNA in patients with active ITP compared to healthy controls. Targeting the immunoproteasome was reported to protect mice from DSS-induced colitis (10) and to attenuate experimental autoimmune encephalomyelitis (EAE) (8). In the present study, passive murine ITP models were treated with ML604440, a highly selective inhibitor of LMP2. ML604440 has not been used *in vivo*. To further characterize the selectivity of ML604440, fluorescent irreversible subunit specific active site probes (Me4BodipyFL-Ahx3Leu3VS) was used according to a previously described method (31) (**Supplementary Figure 2B**). ML604440 failed to revert the thrombocytopenia of model mice. It has been shown that LMP2 inhibition alone does not affect MHC-I cell surface expression, cytokine release, Th17 differentiation, or DSS-induced colitis (32). However, the co-inhibition of LMP2 and LMP7 was previously found to affect these processes and strongly ameliorates disease in experimental colitis and EAE (28). ONX-0914 is a selective LMP7 inhibitor. Basler and colleagues reported that prolonged exposure of cells to ONX 0914 leads to the inhibition of both LMP7 and LMP2 (28). Previous studies have shown that the level of LMP2 is reduced up to approximately 75% in the blood and kidney of arthritis mouse model at 1 h post-intravenous injection of 10 mg/kg ONX-0914 (9). In line with an earlier report, our western blotting results showed that ONX 0914 treatment caused the appearance of LMP7 and LMP2 bands with higher apparent molecular weights.

To investigate the impact of combined LMP2 and LMP7 inhibition on ITP, the effects of ONX-0914 were tested in mice. To our surprise, this inhibitor alleviated thrombocytopenia in passive murine models of ITP. Basler and colleagues reported that the structure, rather than the proteolytic activity, of an immunoproteasome subunit are required for generation of specific epitopes (33). They concluded that inhibition of the catalytic activity of LMP2 by ML604440, which does not affect structural alterations caused by LMP2, has no influence on UTY246–254 presentation. However, experiments using an LMP7-selective inhibitor demonstrated that the catalytic activity of LMP7 was crucial for the production of the UTY epitope (9). Additionally, LMP7 influences the structural features of 20S proteasomes, which thereby enhancing the activity of the LMP2 and MECL-1 catalytic sites (34). Thereby, a single immunosubunit could alter the specificity of the other subunits irrespective of its own hydrolytic activity. Our results indicate that targeting LMP7 and LMP2 with ONX-0914 can alleviated thrombocytopenia in the passive ITP models. Whether blocking of LMP7 or LMP7 and MECL-1 or LMP2 and MECL-1 would have similar effects as observed with LMP7 and LMP2 co-inhibition remains to be determined.

The main mechanism of action in the passive ITP model is antibody-mediated platelet phagocytosis by macrophages (35–37). Functions of macrophages such as phagocytosis and antigen presentation are controlled by the balance between the levels of activating FcγRs (FcgRI, IIa, and III) and that of the inhibitory receptor, FcγRIIb (38). Liu and colleagues reported an increase in CD64 expression, as well in the CD32a/CD32b ratio, in circulating monocytes in ITP patients. HD-DXM or Thrombopoietin receptor agonists (TPO-RAs) therapy shift the FcγR balance towards inhibitory FcγRIIb in the monocytes of patients with ITP (18, 19). However, these authors did not report statistically significant changes in monocyte FcγRIII levels, either in responders or non-responders, after TPO-RA treatment. On the other hand, Zhong and colleagues found that the monocytes of ITP patients exhibit increased expression of CD16 compared to those of healthy controls. Anti-CD16 treatment was previously shown to attenuate thrombocytopenia in ITP patients (39). In this study, we found that ONX-0914 treatment down-regulate FcγRI (CD64) expression in the monocytes of a murine model of passive ITP, and decreased the levels of FcγRIII (CD16) in the monocytes of ITP patients. In addition, ONX-0914 reduced the phagocytosis of antibody-coated platelets by monocyte-derived macrophages of ITP patients *in vitro*. We speculate that the latter event was due to the downregulation of activating FcγRs.

The non-cytotoxic effects of the immunoproteasome inhibition warranted for investigation into the regulatory mechanism. ML604440(300nM) and ONX-0914(30nM) used in our experiments showed no significant cytotoxicity on primary human T cells. Activation markers such as CD69 (early activation marker) and CD25 (late activation marker) are normally expressed when T-lymphocytes are stimulated by antigen binding of T cell receptors (40). A recent study by Chen Y. et al. showed that pediatric ITP patients have particularly higher proportions of CD4+CD25+ T cells than healthy controls (41). Moreover, a previous study from our group showed that CD25 expression is significantly increased in CD4+ T cells and CD8+ T cells of ITP patients compared to controls, indicating that T cells have a higher activation state in ITP patients than in healthy individuals (42). We showed that ONX-0914 decreased activation level of T cells from ITP patients. A study indicated that ONX-0914 impairs T and B cell activation by restraining ERK signaling and proteostais (43). Whether this pathway is affected in ITP by ONX-0914 remains to be determined.

Furthermore, Th cells recognizing platelet antigens and then becoming activated play a crucial role in ITP. An alteration of the CD4+ Th subset has been described in patients with ITP, including increased number of circulating Th1, Th17, and Th22 cells, as well as reduced function of CD4+CD25+FoxP3+ regulatory T cells (Tregs) (44, 45). Among them, Th1 polarization is crucial for the pathogenesis of autoimmune ITP (46, 47), and IFN-γ, which is secreted by Th1, is also important (48). The JAK-STAT pathway plays an important role in IFN-γ-mediated signaling. Phosphorylated STAT1 is an important signal mediator that translocates to the nucleus and activates the transcription of target genes, including IFN-γ. A specific single-nucleotide polymorphism in *STAT1*, rs1467199, plays a potential role in IFN-γ dependent autoimmunity in pediatric ITP (49, 50). Recently, several studies have shown that ONX-0914 blocked production of T cell–mediated IFN-γ production in human PBMCs and in Hashimoto’s model (9, 51). A lower phosphorylation of STAT1 upon ONX-0914 even after 1 h of Th1-polarizing conditions, thus pointing toward a direct role of ONX-0914 in driving Th1 differentiation (52). Our studies demonstrated that ML604440 does not affect the activation or differentiation of Th cells. In addition to suppressing the activation of CD4+ T cells, the co-inhibition of LMP2 and LMP7 by ONX-0914 blocked Th1 differentiation and inhibited STAT1 phosphorylation. We agree with a hypothesis that immunoproteasome inhibition might be stabilizing a phosphatase, which is specifically degraded by the immunoproteasome subunit and in turn leading to the effects of reduced phosphorylation on Stats. Further studies are necessary to identify these putative phosphatases, which are influencing different Th cell differentiation pathways (52).

In conclusion, our findings revealed that the mmunoproteasome may play a pathogenic role in ITP. Co-inhibition of LMP2 and LMP7 inhibited macrophage phagocytosis, downregulated FcγI in ITP mice, decreased FcγIII in ITP patients, inhibited T cell activation and Th1 differentiation, and alleviated thrombocytopenia in the passive ITP models. In conclusion, the combined inhibition of LMP2 and LMP7 has the potential to become a novel therapeutic strategy for ITP patients.

## Data Availability Statement

The datasets presented in this study can be found in online repositories. The names of the repository/repositories and accession number(s) can be found in the article/**Supplementary Material**.

## Ethics Statement

The studies involving human participants were reviewed and approved by Medical Ethical Committee of Qilu Hospital. The patients/participants provided their written informed consent to participate in this study. The animal study was reviewed and approved by Medical Ethical Committee of Qilu Hospital.

## Author Contributions

YS designed and funded the research. S-HD and Y-JX performed the research and contributed equally to this study. LL, MN, and YH assisted the research. LW and B-BL analyzed the data. S-HD wrote the paper. YS, MH, JP, Q-LT, and MX edited the paper. All authors contributed to the article and approved the submitted version.

## Funding

This work was supported by grants from the National Natural Science Foundation of China (81170475, 81470285, 81770114, 81770133, 81470284), Major Research Plan of National Natural Science Foundation of China (91442204), Natural Science Foundation of Shandong Province (ZR2017PH022, ZR2017PH041) and Major Research Plan of Natural Science Foundation of Shandong Province (ZR2016QZ008).

## Conflict of Interest

The authors declare that the research was conducted in the absence of any commercial or financial relationships that could be construed as a potential conflict of interest.
