# Supplementary material for: Co-Inhibition of the Immunoproteasome Subunits LMP2 and LMP7 Ameliorates Immune Thrombocytopenia
Source: Front Immunol. 2021 Jan 20;11:603278. doi: 10.3389/fimmu.2020.603278 (PMC7855704; doi:10.3389/fimmu.2020.603278)
Supplement: Supplementary file 1 [file DataSheet_1.docx]

**Supplemental Methods**

**Profiling of Proteasome Activity Using SDS-PAGE Based Assays**

1. Harvest the PBMCs from ITP patients which treated with DMSO or 300 nM ML604440 for 72 h or splenocyte from passive ITP mice after treated daily with 10mg/kg ML604440. Transfer cells to a Falcon tube and centrifuge at 1,200×g for 2 min at 4°C. Discard the supernatant and resuspend the cell pellet in 10–20 pellet volumes of PBS. Transfer cells to an Eppendorf tube and pellet cells by centrifugation at 1,200×g for 2 min at 4°C. Discard the supernatant.

2. Resuspend cells in 2 pellet volumes of cold HR buffer(50 mM Tris–HCl (pH 7.4), 5 mM MgCl2 , 250 mM sucrose, 1 mM DTT (added fresh from a 1 M stock solution before use), 2 mM ATP (added fresh from a 0.5 M stock solution before use). Prepare HR buffer lacking DTT and ATP, filter over a 0.22 mm filter (e.g., MILLEX®GS, Millipore), and store at 4°C. Supplement the amount of HR buffer needed for a single experiment (typically 1 mL) with ATP and DTT before use). Lyse cells mechanically Centrifuge for at 14,000×g for 3 min at 4°C to remove membrane fractions and cell debris and transfer the supernatant to a fresh Eppendorf tube.

3. Determine protein concentrations using a Bradford assay.

4. To label proteasome subunits directly, transfer 25 mg lysate to a fresh Eppendorf tube and adjust the volume to 24.5 mL with HR buffer. Add 50 mM Me4 BodipyFL-Ahx3 Leu3 VS stock solution in DMSO to obtain a final probe concentration of 1 mM and a final protein concentration of 1 mg/mL. Vortex and incubate the sample for 1 h at 37°C.

5. These instructions assume the use of the NuPAGE precast gel system (Invitrogen) and precast mini gels to separate proteins on SDS-PAGE.Add 12.5 mL 3× reducing sample buffer to each sample. The volume of these samples should be 25 mL. Vortex and denature by boiling the sample for 10 min at 70°C. Centrifuge at 14,000×g for 1 min at room temperature.

6. Assemble the NuPAGE gel unit using a precast NuPAGE 12% Bis-Tris gel according to the manufacturer’s instructions. Add 1× MOPS buffer to both the inner and outer chamber of the gel unit. Add 125 mL antioxidant to the inner gel chamber only. Load 10 mL of denatured sample per well. Keep one well free and load this well with 6.5 mL prestained molecular weight marker (e.g., SeeBlue® Plus2 Pre-Stained Standard from Invitrogen). Load 3 mL of 3× reducing sample buffer to any remaining wells.

7. Run the gel at 90 V until the15 kDa protein in the molecular weight marker (Lysozyme in the SeeBlue® Plus2 Pre-Stained Standard) is at the bottom of the gel.

8. Remove the gel from the cassette and image the wet gel slab for 10–120 s using a fluorescence imager(Amersham Imager 600，General Electric) containing an appropriate filter set (excitation at 480 nm, emission at 530 nm).

9. Analyze images using appropriate software to quantify fluorescence intensities.

**Effects of Immunoproteasome Inhibitors on T Activation in ITP mice**

After receiving ONX-0914(10mg/kg) treatment for 6 days for the passive ITP model, mice were sacrificed. Peripheral blood and spleens were harvested. Single cell suspensions were prepared. Cells were treated with hemolysis buffer to remove red blood cells. After washing in PBS, we counted cells and split them. For measuring the expression of CD25, cells were incubated with APC anti-mouse CD25 (Biolegend)and FITC anti-mouse CD4 (Biolegend) in the dark at Room temperature(RT) for 30 min, washed with cold PBS, and analyzed by ﬂow cytometry within 1 h.

**Supplemental Figures**

**
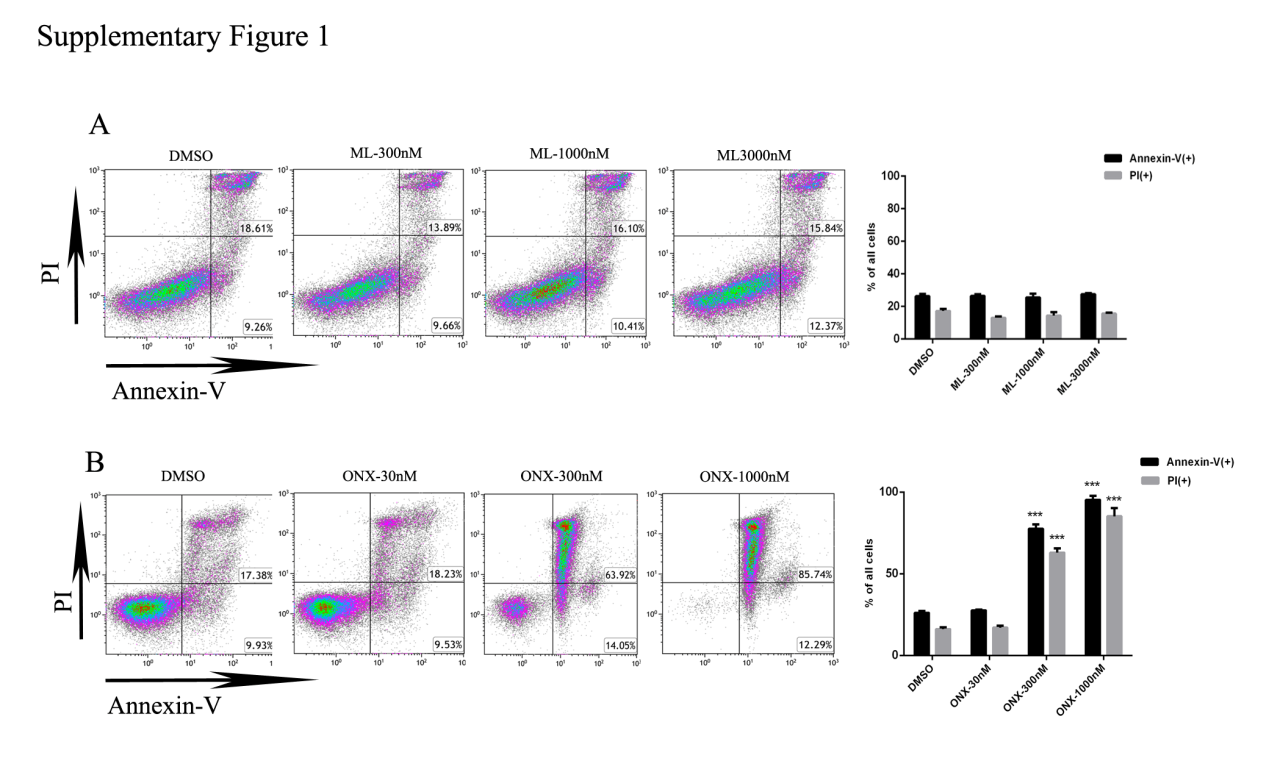
**

**Supplementary Figure 1. ML604440 is not cytotoxic to primary human cells but ONX-0914 is**. PBMCs from ITP patients were treated with DMSO or different concentrations of ML604440 (A) or different concentrations of ONX-0914(B) for 72h and then stained with AnnexinV and PI. Signals were detected by flow cytometry. Shown are the quantification of early apoptotic (AnnexinV+) or dead (PI+) cells from the whole cell population (n = 6; ***p < 0.001).


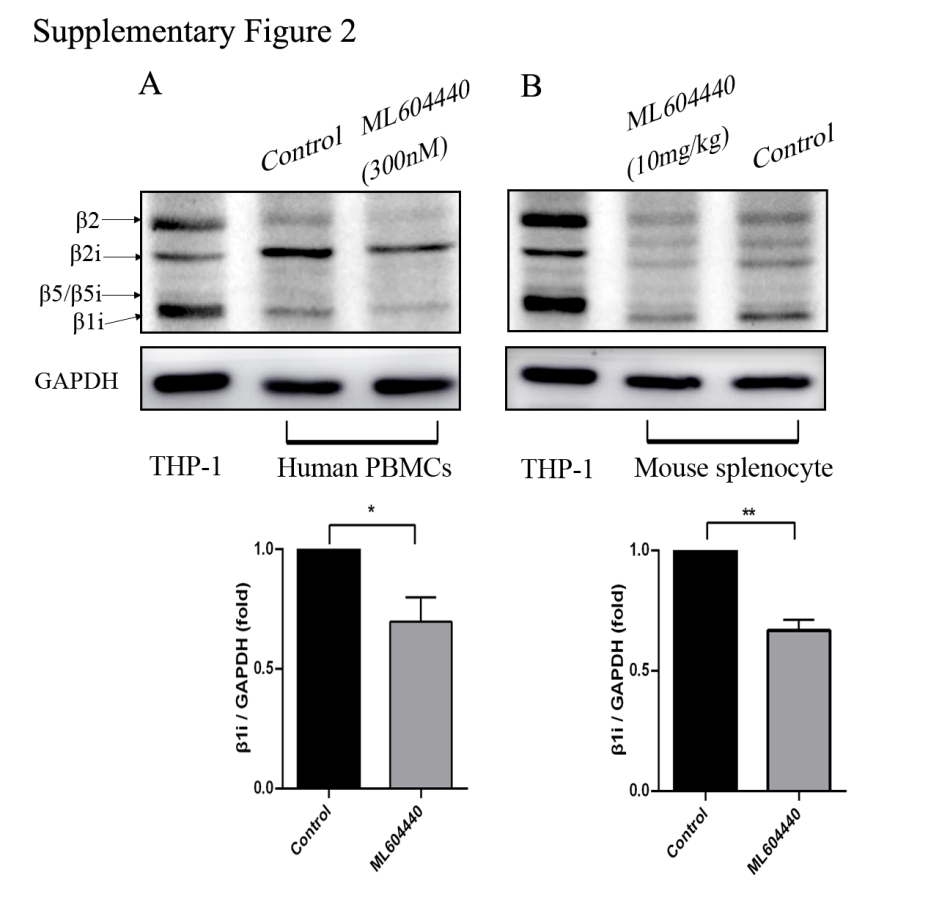


**Supplementary Figure 2.** **Gel images showing the active proteasome subunit labeling in PBMCs from ITP patients and splenocyte from mice cell lysates.** (A) PBMCs from ITP patients were treated with DMSO or 300 nM ML604440 for 72 h, or (B) the passive ITP mice treated daily with 10mg/kg ML604440 for 6 days. cell lysates of PBMCs or splenocyte from mice were incubated with 1 mM Me4 BodipyFL-Ahx3 Leu3 VS, proteins were separated by SDS-PAGE and the resulting gel was scanned for fluorescence emission. The densitometry analysis of β1i （LMP2）from three independent experiment shown above. Un-treated THP-1 (Human monocytic leukemia) cells’ all active subunits are labeled, and the composition of proteasome in the cells was visualized. THP-1 cells play a role in marking molecular weight.


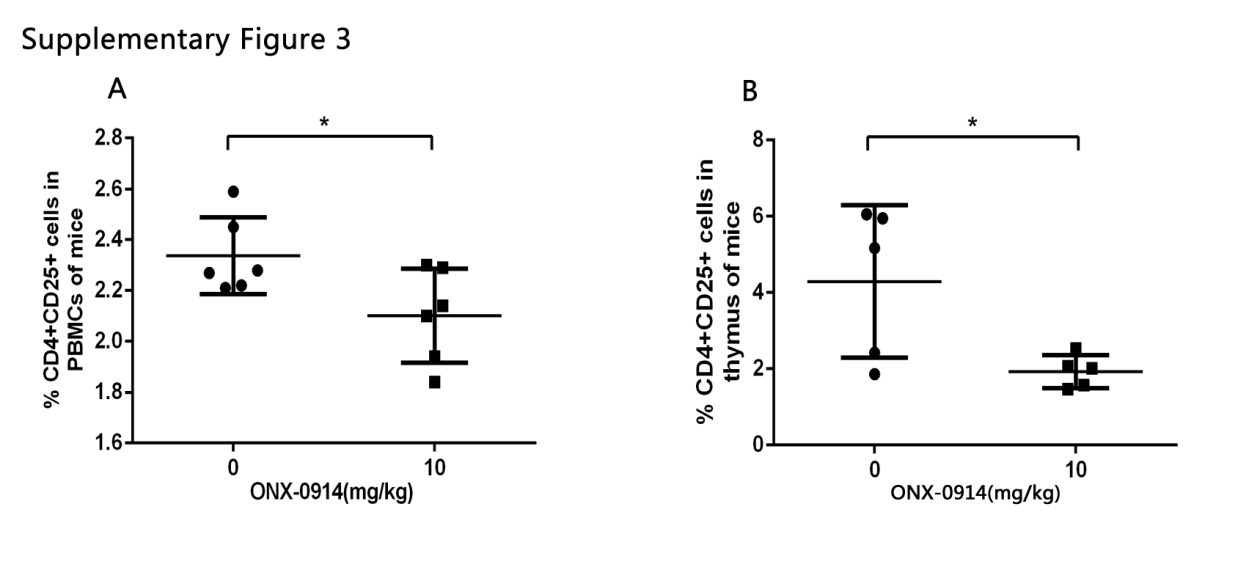


**Supplementary Figure 3. ONX-0914 decreased CD4+CD25+ T cells from immune thrombocytopenia (ITP) mice.** In PBMCs and thymus of ITP mice, % CD4^+^CD25^+^ T cells were lower in the ONX-0914 (10 mg/kg) group than in the control group. (n=6;* p<0.05)
